# The clear success of hip preservation

**DOI:** 10.1093/jhps/hnu001

**Published:** 2014-05-22

**Authors:** Richard Villar

**Affiliations:** Editor-in-Chief

Another journal? Surely not. Yet of course another journal is needed. It is manifestly and glaringly obvious. But I can see why some may question the reason. After all, by 2009 there were known to be more than 25 400 scholarly peer-reviewed journals in the scientific, technical and medical (STM) field, producing about 1.5 million articles each year [1] These numbers grow by ∼3.5% and 3% per annum, respectively—a growth rate that has not changed for three centuries [2]. Why add to that load?

Yet reading patterns are changing, and although researchers read more than they ever did—approximately 270 articles each year—they spend less time physically at the task. In the mid 1990s, the mean reading time for a single article was 45–50 min, a figure that has reduced to ∼30 min now [3, 4]. This is quite in contrast with the length of time it can take to prepare a typical scientific paper, which is estimated to be as much as 90–100 h [5]. Meanwhile, the cash cost of producing a single, hard copy article in a subscription-only journal has been estimated at £2863 (US$4804) in the United Kingdom [6]. This has since been revised upwards to £3095 (US$5193) [7]. Moving to an online-only format can save up to 20% of these costs, clearly an interesting development in this era of declining advertising revenue. The way in which we read has also changed, with more emphasis on searching and less on browsing. No longer are we content to fall asleep over our monthly issue of an association’s high-profile general orthopaedic journal.

Into this mix comes open access (OA), the process of making original research articles freely accessible to readers on the web. There is now a global shift in this area with a study funded by the European Commission, suggesting that OA has reached a critical tipping point with ∼50% of scientific papers published in 2011 now being available for free [8]. Furthermore, the European Commission has instructed that as of 2014, all articles produced with funding from Horizon 2020 [9]—the next European Union research and innovation funding programme—will have to be made immediately accessible online by the publisher. Failing that, it must be made available by the researcher through an OA repository no later than 6 months after publication [10].

The US White House appears to be going down a similar route. The Office of Science and Technology Policy has directed each federal agency that spends more than US$100 million each year on research and development to support increased public access to the results. This is a major shift in policy. The intent is to use a 12-month post-publication embargo period as a guideline for making research papers publicly and freely available [11–13].

Criticisms of OA, certainly in the past, have been the quality of the peer review and a lack of the prestige required by academics should they publish. The so-called article processing charge (APC), where the author might pay for publication, has also been a worry. An APC might be sought on either submission or acceptance. Yet such a worry seems strange. Very little in life is for free, and publishing is no exception. Even subscription-only journals must recoup their costs, although traditionally do so at the reader, rather than the author end of the spectrum. The costs are perhaps more hidden—maybe as part of an association’s membership fee, possibly a library subscription, or even requesting author payment for colour images. Indeed, some well-established subscription journals have chosen to charge their authors a paper-submission fee anyway, so there is a balancing process between subscription and OA journals presently underway. Already, results indicate that OA journals indexed in *Web of Science* and/or *Scopus* are approaching the same scientific impact and quality as subscription journals, particularly in biomedicine and for journals funded by APCs [14].

Authors’ perceptions of journals also change over time. Publications that were once specialist are now regarded as generalist. Indeed in those distant days when surgery of the musculoskeletal system formed part of a general surgeon’s repertoire, orthopaedic surgery itself was seen as a subspecialty area. Now it, too, has become subdivided into many subspecialties.

The development of journals, numerous though they are, appears to parallel changes in medicine and surgery. New techniques emerge, some successful, some not. For those that succeed, research naturally amasses and journals surely follow. So it is no surprise that this new journal, *Journal of Hip Preservation Surgery (JHPS)*, has been welcomed with open arms. It is testament to the success of a rapidly developing subspecialty field.

For many the journal’s arrival is not before time as the art—yes, let us call it an art—of hip preservation is developing a huge momentum. Position those two words, hip preservation, into an indexing system, and almost instantly 826 references will appear [15]. Take 100 of these, and they will be spread across 40 journals; 25 of these articles are OA. Forty journals is simply too much reading for any one individual; so to bring the best of that information between the online covers of *JHPS* makes perfect sense. Never has the timing been better for a new journal to appear, never has the choice of subspecialty been so apt and never has an interest been so keen.

Welcome then to this, the first editorial for our new baby, *JHPS*. Remember that this journal was created for you, in many respects by you, evidence of the clear success of hip preservation.
